# Rare Pulmonary Granular Cell Tumor Presenting as an Endobronchial Lesion: A Case Report

**DOI:** 10.7759/cureus.80471

**Published:** 2025-03-12

**Authors:** Abolfazl Sodagar, Sujeirys Paulino, Nismat Javed, Marcos Molina, Misbahuddin Khaja

**Affiliations:** 1 Internal Medicine/Pulmonary Critical Care, BronxCare Health System, Bronx, USA; 2 Internal Medicine, BronxCare Health System, Icahn School of Medicine at Mount Sinai, New York, USA; 3 Internal Medicine, BronxCare Health System, Bronx, USA; 4 Internal Medicine/Pulmonary Critical Care, BronxCare Health System, Icahn School of Medicine at Mount Sinai, New York, USA

**Keywords:** benign, granular cell tumor, management, outcome, pulmonary granular cell tumor

## Abstract

Granular cell tumors (GCTs) are rare soft tissue tumors with a myogenic origin. While GCTs typically arise in the tongue, skin, and subcutaneous tissues, pulmonary involvement (pGCT) remains rare. The most common site in the case of pGCT is the bronchus, followed by the trachea and lung parenchyma. In this case, we discuss the presentation of a 33-year-old female with respiratory distress who was subsequently diagnosed with pGCT, and her dyspnea resolved after conservative management. Bronchoscopic evaluation and biopsy with histopathological examination remain the cornerstone for diagnosis and management. While most pGCTs are benign, a few can be malignant and require additional techniques for management. Given their infrequency, pGCTs can pose diagnostic challenges, often mimicking other endobronchial lesions clinically and radiologically. Management strategies range from conservative observation especially for smaller, asymptomatic tumors to surgical resection or bronchoscopic excision for those exhibiting significant symptoms, airway compromise, or features suggestive of malignancy.

## Introduction

Granular cell tumors (GCTs) were first recognized by Abrikossoff in 1926 and labeled "myoblastomas" based on the belief they originated from myoblasts. Several years later, in 1935, Feyrter introduced the term "granular cell neuroma," suggesting a neural origin. Immunohistochemical evaluations subsequently identified Schwann cells as the most likely source, supported by structural similarities and the presence of markers such as S-100, myelin proteins PO and P2, and enolase [1-3]. Although GCTs most commonly arise in the tongue, skin, and subcutaneous tissues, pulmonary GCTs account for only 2-6% of cases, primarily affecting the bronchus (78.3%), followed by the trachea (12.5%) and lung parenchyma (9.1%) [4]. While the majority of pulmonary GCTs (pGCTs) are benign, 1-3% exhibit features of malignancy, including spindling, pleomorphism, necrosis, and elevated mitotic activity [5]. This case report focuses on a benign pGCT and discusses relevant diagnostic and management approaches drawn from the literature.

## Case presentation

**Figure 1:**
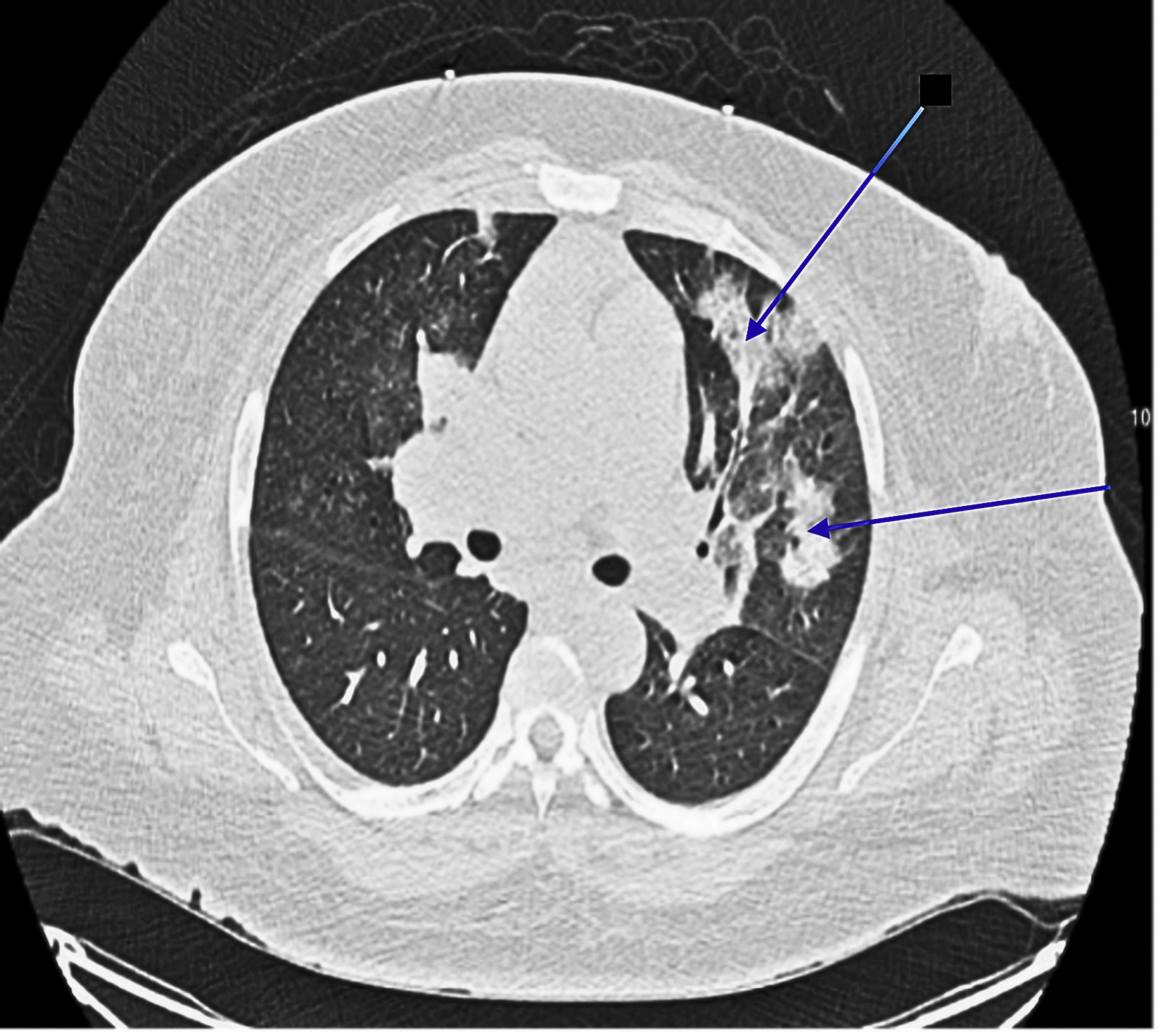
CT scan showing left-sided opacifications (marked by blue arrows in the axial view)

**Figure 2:**
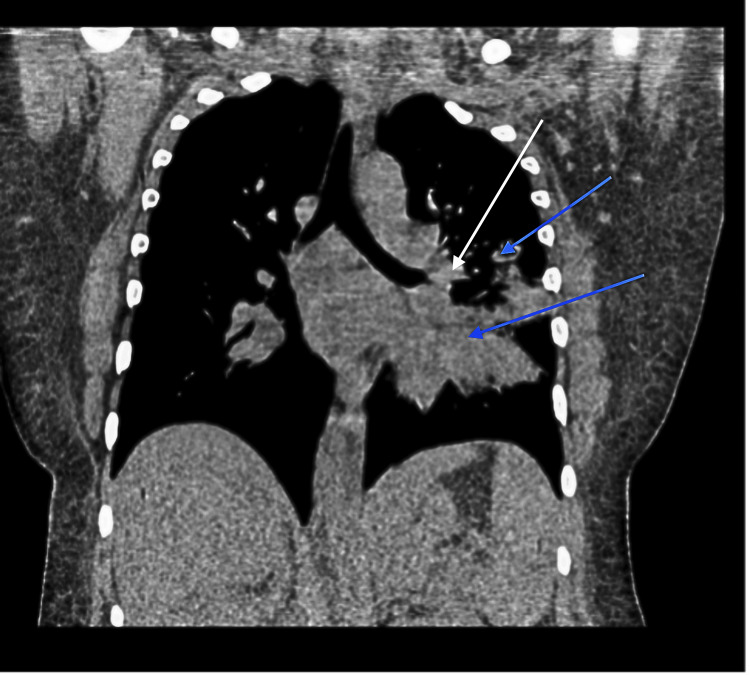
Coronal view of the CT scan showing multifocal pneumonia within the left upper and lower lobes (marked by blue arrows) and a left hilar opacity (marked by the white arrow)

A 33-year-old female with a history of liver cirrhosis and tobacco use disorder presented to the hospital with acute respiratory distress, hemoptysis, and persistent cough. Physical examination revealed tachypnea and decreased breath sounds over the left hemithorax. Laboratory studies were significant for mild leukocytosis. Computed tomography (CT) of the chest demonstrated multifocal pneumonia and a left hilar opacity (Figures 1, 2).

**Figure 3:**
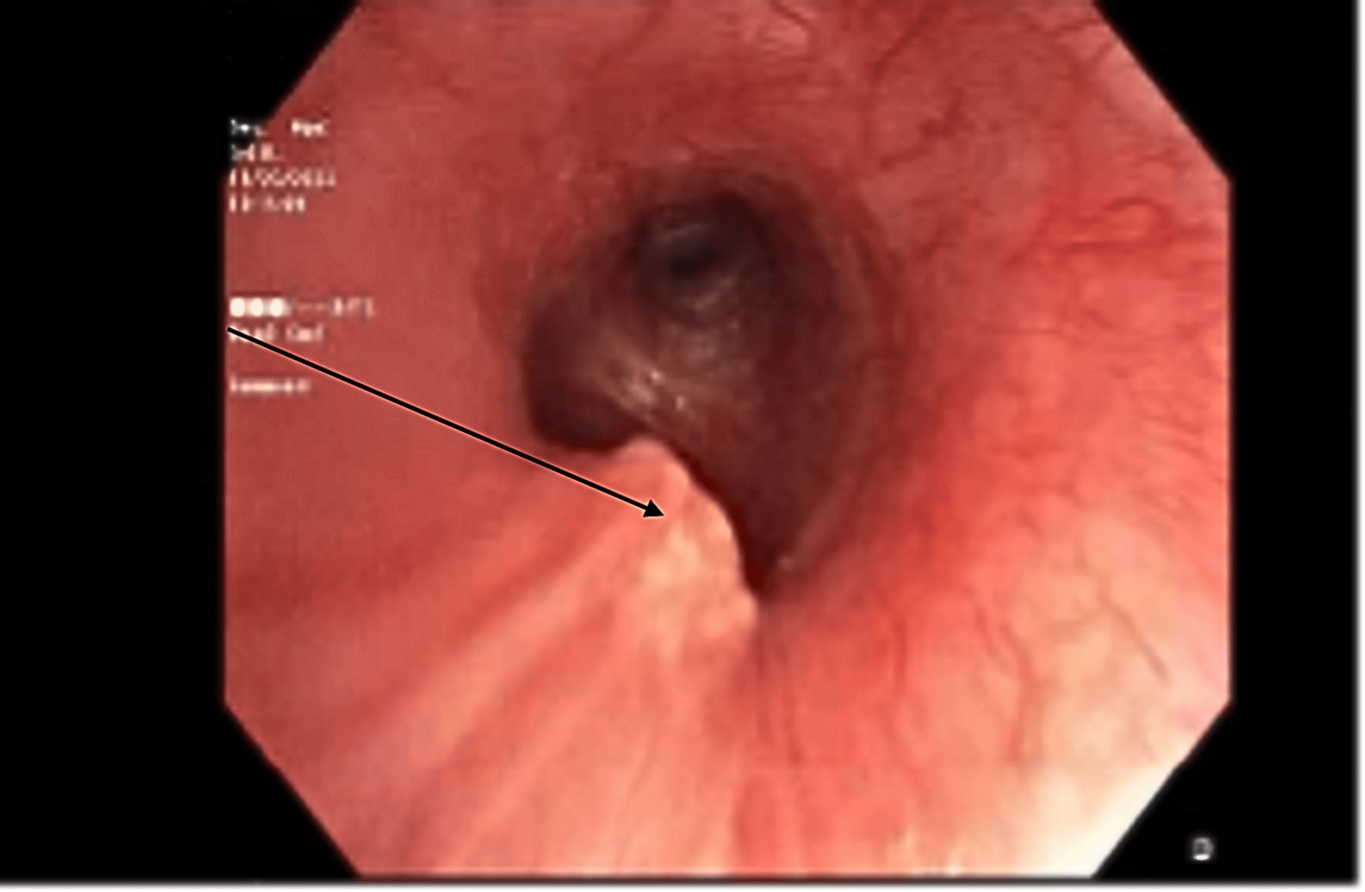
Fiberoptic bronchoscopy showing an endobronchial lesion in the left main bronchus (black arrow)

She was started on empiric antibiotic treatment for pneumonia and admitted to the intensive care unit for respiratory monitoring. Due to worsening respiratory status, the patient required intubation with mechanical ventilation. Bronchoscopy revealed a polypoid endobronchial lesion in the left main bronchus, approximately less than 1 cm, partially obstructing airflow, and was biopsied (Figure 3). 

**Figure 4:**
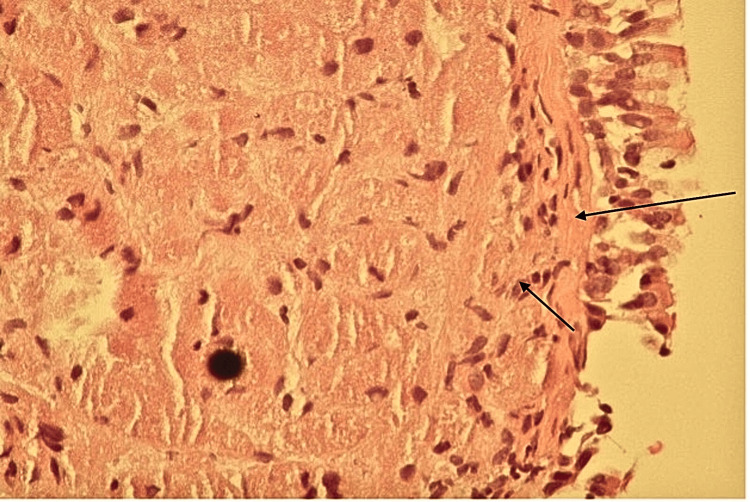
Histopathology showing sheets of granular cell tumor cells in the submucosa (black arrows)

The examination of the biopsy specimen revealed large polygonal cells with granular, eosinophilic cytoplasm and small, round nuclei (Figure 4). Immunohistochemical staining was positive for S-100, neuron-specific enolase (NSE), and vimentin, confirming the tumor's Schwann cell origin, unlike myogenic tissue as expected.

The patient was managed conservatively with antibiotics and supportive care. Later, her symptoms resolved, and she was discharged with plans for ongoing imaging surveillance to monitor for recurrence or progression.

## Discussion

pGCTs are uncommon. They account for roughly 0.5% of all soft tissue tumors, most frequently originating in the bronchus and presenting as polypoid lesions during endoscopy [1,4,6]. Multifocal disease occurs in 4-10% of cases but does not necessarily indicate malignancy. Up to 40% of pGCTs demonstrate local infiltration into peribronchial tissue, though lymph node involvement remains rare [2]. These tumors are more prevalent among women (63%) and typically occur in individuals aged 30-50 years. Approximately one-third of patients have a history of smoking [2]. A literature review from 1980 to 2024 revealed seven adult cases of pGCTs reported (Table 1) [7-12].

**Table 1:** Summary of cases reviewed BAL: bronchoalveolar lavage

Author	Age/Gender	Symptoms	Imaging	Treatment and Histopathology	Outcome
Radić et al. [7]	49/F	2-month history of fever and cough	CT: infiltrating mass of the right upper lobe, peribronchial infiltration, and pronounced air bronchogram. Fiber bronchoscopy revealed obstruction of the right upper lobe bronchus.	Right-sided lobectomy Histopathology: polygonal cells with granular cytoplasm and small nuclei. All tumor cells were S-100 positive immunohistochemically, while CD68 staining demonstrated weak, focal positivity.	The patient was alive. No recurrence after 11 years.
Radić et al. [7]	55/M	Long-standing history of heart failure exacerbations	Chest X-ray: right right-sided pleural effusion. Cytology showed reactive cells with lymphocytes and granulocytes. Bronchoscopy showed some infiltration of the right upper lobe of the lung and altered mucosa of the main bronchus.	Endoscopic removal Histopathology revealed metaplastic, reactive squamous epithelium. Cells were strongly positive for S-100 and CD68.	The patient was alive. No recurrence over three years.
Kim et al. [8]	77/F	Chest discomfort and dyspnea	Flexible bronchoscopy revealed total occlusion of the right main bronchus by the mass. CT: a calcified mass extending to the right main bronchus with origin in the bronchus intermedius, lymph node involvement was noted.	No surgical intervention. Histopathology showed polygonal cells and pleomorphic nuclei with occasional prominent nucleoli concerning malignancy. The tumor cells were diffusely positive for CD68 and TFE-3 and negative for S-100, suggesting non-neural malignant GCT.	Planned for chemotherapy and radiotherapy but was lost to follow-up.
Matthew et al. [9]	34/F	Respiratory distress requiring intubation and tracheostomy	Chest X-ray showed multiple infiltrates. Bronchoscopy was done for checking placement of the tracheostomy, and an endobronchial mass obstructing the right middle lobe was noted.	Ablation, alternated with debulking. Pathology revealed a granular cell tumor.	The patient was decannulated, had no recurrence, and was discharged.
Meena et al. [10]	31/M	Asymptomatic	CT scan showed a mass. On bronchoscopy, there were two separate but similar endobronchial lesions in the distal trachea and at the right upper lobe bronchus level.	Subsequent rigid bronchoscopy with ablation. Histopathology revealed a granular cell tumor.	The patient was alive and had no recurrence.
Lauro et al. [11]	61/M	Chronic cough	CT scan showed an unresectable left bronchial mass. Bronchoscopy revealed an endobronchial mass. A repeat CT scan showed a 30% increase in the diameter of the lesion.	Initial histology showed a granular cell tumor. After 3 weeks, repeat biopsy based on the CT scan lesion revealed the presence of small-cell lung cancer. Underwent six cycles of cisplatin and etoposide.	The patient was alive, and the CT scan showed complete disappearance of the neoplasm. The bronchoscopy examination showed no endobronchial lesion.
Endicott-Yazdani et al. [12]	55/F	Chronic dyspnea	Chest X-ray and CT scan showed a right lobar opacity. Bronchoscopy with BAL was performed, and multiple pale submucosal endobronchial masses were observed. One large lobulated mass caused 90% obstruction.	Debulking biopsy. Endobronchial biopsy specimens showed submucosal proliferation of cells with granular eosinophilic cytoplasm and positive S100 staining.	The patient was lost to follow-up.

Most of the patients had a relatively benign course. However, one patient developed another malignancy that warranted oncological treatment [11]. In most cases, patients had symptoms predominantly of chronic cough [7,11,12], except for one asymptomatic patient [10]. As noted in all cases, bronchoscopy and biopsy were crucial to diagnosis [7-12]. While surgical interventions, such as excision or debulking biopsy, were frequently performed, a conservative approach was adopted for our patient due to the size and symptoms experienced [7-12]. The patient's tumor was managed conservatively due to the absence of significant airway compromise and lack of invasive features, which was suggestive of a benign nature.

pGCTs are distinguished histologically by polygonal cells with abundant eosinophilic cytoplasm due to the presence of cytoplasmic lysosomes [5]. Immunohistochemical staining of benign pGCTs is consistently positive for S-100, NSE, vimentin, and CD68 [5]. In contrast, malignant variants exhibit spindling, elevated mitotic figures, necrosis, and a high nuclear-to-cytoplasm ratio. Increased expression of p53 and Ki-67 is observed in malignant cases, with Ki-67 positivity exceeding 30% in aggressive tumors [5]. Radiologically, pGCTs typically appear as well-defined endobronchial masses, and positron emission tomography (PET) scans often reveal minimal or no FDG uptake, helping differentiate these lesions from malignancies such as carcinoid tumors [2]. Bronchoscopy remains the diagnostic gold standard by allowing direct visualization and tissue sampling for histopathological confirmation [3].

Clinical features, including weight loss and hemoptysis, might help differentiate benign from malignant lesions. Imaging studies reveal a higher growth rate and the presence of metastasis, which are features that might differentiate malignant from benign tumors. However, the standard remains histopathological diagnosis. Due to their rarity and limited studies, diagnosing malignant GCT remains challenging. In 1998, Fanburg-Smith et al. established histological criteria to classify GCTs as malignant, atypical, or benign based on six features: nuclear pleomorphism, tumor cell spindling, vesicular nuclei with large nucleoli, increased nucleus-to-cytoplasm ratio, necrosis, and a high mitotic rate (>2 mitoses per 10 high-power fields) [13]. Tumors exhibiting more than three of these traits were deemed malignant while those with one or two were considered atypical, and those with none or only focal pleomorphism were classified as benign. Nasser et al. proposed a classification based on necrosis and mitotic activity, categorizing tumors with either feature as having uncertain malignant potential [14]. At the same time, metastasis alone was sufficient for malignancy. Comparatively, benign lesions would not have these criteria.

Management of pGCTs hinges on tumor size, location, and symptomatology. Small, asymptomatic lesions (<1 cm) can be observed or excised via endoscopic methods such as bronchoscopic excision or laser therapy [15]. Larger tumors (>1 cm) or those with significant local invasion generally warrant more extensive surgical resection, including lobectomy or sleeve resection, to ensure complete removal. While benign pGCTs are often curable through surgical excision, malignant variants carry a heightened risk of recurrence and metastasis [4]. Notably, lesions greater than 1 cm in diameter are prone to involving the entire thickness of the tracheal wall, making bronchoscopic removal less effective and favoring a surgical approach [16]. Although surgical resection can be curative, recurrences may still occur, so follow-up for at least five years is recommended [2,3].

## Conclusions

Pulmonary granular cell tumors are uncommon neoplasms that require a comprehensive diagnostic workup to guide appropriate treatment. Histological and immunohistochemical analyses play a pivotal role in differentiating benign from malignant variants. Management should be tailored to each patient's clinical scenario and tumor characteristics: while small, asymptomatic lesions may be managed conservatively, more extensive or invasive tumors usually require surgical intervention. Long-term monitoring is essential to ensure favorable outcomes and to promptly detect any recurrence or disease progression.
